# Fe_3_O_4_@SiO_2_ nanoparticle supported ionic liquid for green synthesis of antibacterially active 1-carbamoyl-1-phenylureas in water

**DOI:** 10.1039/c8ra04368j

**Published:** 2018-08-02

**Authors:** Mahmoud Nasrollahzadeh, Zahra Issaabadi, S. Mohammad Sajadi

**Affiliations:** Department of Chemistry, Faculty of Science, University of Qom Qom 3716146611 Iran mahmoudnasr81@gmail.com +98 25 32103595 +98 25 32850953; Department of Petroleum Geoscience, Faculty of Science, Soran University PO Box 624 Soran Kurdistan Regional Government Iraq

## Abstract

In the present work, we have designed a novel, heterogeneous and recyclable magnetic Brønsted acidic ionic liquid based on 5-phenyl-1*H*-tetrazole. The {Fe_3_O_4_@SiO_2_@(CH_2_)_3_5-phenyl-1*H*-tetrazole-SO_3_H/Cl} ([FSTet-SO_3_H]Cl) was prepared *via* the immobilization of 5-phenyl-1*H*-tetrazole-bonded sulfonic acid onto the surface of silica-coated magnetic nanoparticles using 3-chloropropyltriethoxysilane as a linker. The catalyst was characterized by XRD, TEM, FESEM, EDS, TG-DTA, and FT-IR. The ability and high activity of this catalyst were demonstrated in the synthesis of 1-carbamoyl-1-phenylureas with good to excellent yields *via* a new, simple and one-pot procedure in aqueous media under reflux conditions. This procedure has advantages such as high yields, short reaction times, a simple methodology and work-up process, green reaction conditions, high stability, catalytic activity, and easy preparation, separation and reusability of the catalyst. The synthesis of these compounds was confirmed by FT-IR, ^1^H NMR, ^13^C NMR and CHN. In addition, we investigated the biological properties of the 1-carbamoyl-1-phenylureas as newly synthesized compounds. The described catalyst could be easily separated from the reaction mixture by additional magnetic force and reused several times without a remarkable loss of its catalytic activity and any considerable changes in the product yield and the reaction time.

## Abstract 2

We have developed a novel and green method for the synthesis of antibacterially active 1-carbamoyl-1-phenylureas in water using {Fe_3_O_4_@SiO_2_@(CH_2_)_3_5-phenyl-1*H*-tetrazole-SO_3_H/Cl} ([FSTet-SO_3_H]Cl) as a recyclable magnetic catalyst.

## Graphical abstract

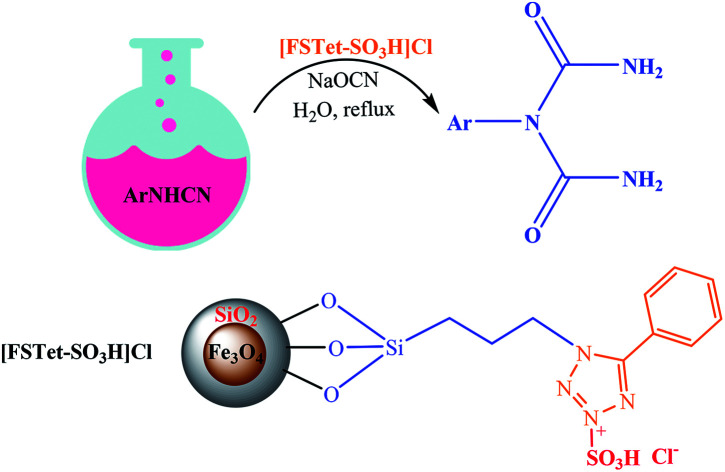

## Introduction

Ionic liquids (IL) are a class of liquids that contain only ions.^1^ In general, ionic liquids contain molten salts at a temperature above 800 °C, however today, ionic liquids are known as salts that are liquid at temperatures below 100 °C.^2^ Over the past few years, ionic liquids have attracted the attention of many scientists due to their capabilities as catalysts, reaction media, reagents and solvents.^3^ Ionic liquids have advantages such as a high thermal stability, being non-flammable, having a low vapor pressure, being recyclable and having excellent solvation properties.^4^ However, ionic liquids have limited applications due to their toxic nature.^5^ Recently, extensive studies have been conducted to eliminate the disadvantages associated with ionic liquids and replace them with safer and more productive ones. One of the best techniques is the combination of ionic liquids and magnetic nanoparticles (MNPs). MNPs act as a good support for the immobilization of the ionic liquids.^6^ Magnetic ionic liquids have certain specifications such as a large specific surface area, high stability, facile separation and recovery from the reaction mixture, good magnetic permeability and low toxicity and price.^7^

Recently, solid acid catalysts have attracted a lot of attention in the field of organic reactions.^8^ In this regard, many Brønsted acids such as thiourea, TADDOL, amidinium and phosphate can be used as green and free-of-metal catalysts.^9^ If an alkane sulfonic acid group is covalently tethered to the IL cation, the IL is converted into a strong Brønsted acid.^10^ These SO_3_H-functionalized ionic liquids can act as good alternatives for homogenous and heterogeneous acidic catalysts due to their advantages such as being non-corrosive, nonvolatile and immiscible with many organic solvents.^11^

From the past to present, the synthesis of organic compounds has attracted the attention of many chemists due to their special importance in biological and medical studies. Among the organic compounds, 1-carbamoyl-1-phenylureas are an important class of compounds. However, there is no report on the synthesis of 1-carbamoyl-1-phenylureas in literature so far.

Over the past few years, the hydration of cyanamides has attracted a lot of attention as one of the most important ways of synthesizing *N*-monosubstituted ureas.^12^ However, the hydration of cyanamides suffers from several disadvantages including the use of corrosive bases or acids, low yields, the use of toxic organic solvents, long reaction times and tedious work-up.^13^ Therefore, the development of a new, easy and efficient method for the hydration of cyanamides is one of the most important challenges.

1-Carbamoyl-1-phenylureas are the parent compounds of a large and interesting class of organic substances. It is probable that the presence of three nitrogen atoms in their structure has led to greater biological properties, but still nobody has succeeded in synthesizing this important compound. In addition, they are important compounds which can be used as starting materials in coordination chemistry and organic synthesis in the future.

**Scheme 1:**
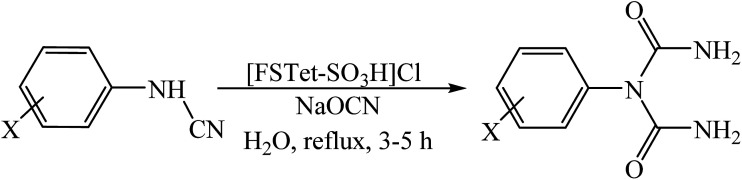
Synthesis of 1-carbamoyl-1-phenylureas.

In this research, we have designed a heterogeneous and recyclable magnetic Brønsted acidic ionic liquid catalyst by using 5-phenyl-1*H*-tetrazole. Although many ionic liquid based imidazoles are known, only very few ionic liquid based tetrazoles have been described.^14^ However, it is noteworthy that there is no report on the synthesis of magnetic ionic liquid based tetrazoles. Therefore, this report could create a new approach for the production of magnetic ionic liquid based tetrazoles. Next, we investigated the catalytic activity of [FSTet-SO_3_H]Cl in the synthesis of 1-carbamoyl-1-phenylureas *via* a one-pot procedure in aqueous media under reflux conditions (Scheme 1). The results show that the catalyst has excellent catalytic activity in this reaction. The products were prepared in good to excellent yields and characterized by FT-IR, ^1^H NMR, ^13^C NMR, CHN and melting point determination.

## Experimental

### Reagents and methods

All materials of commercial reagent grade were purchased from the Merck and Aldrich companies and used without further purification. FT-IR spectra were recorded on a Nicolet 370 FT/IR spectrometer (Thermo Nicolet, USA) using pressed KBr pellets. X-ray diffraction (XRD) measurements were carried out with a Philips powder diffractometer type PW 1373 goniometer. It was equipped with a graphite monochromator crystal. The X-ray wavelength was 1.5405 Å and the diffraction patterns were recorded in the 2*θ* range (10–80) with a scanning speed of 2° min^−1^. TEM images were taken using a Philips EM208 transmission electron microscope with an accelerating voltage of 90 kV. Scanning electron microscopy (SEM) of the {Fe_3_O_4_@SiO_2_@(CH_2_)_3_5-phenyl-1*H*-tetrazole-SO_3_H/HCl} was performed on a Cam scan MV2300. The chemical compositions of the synthesized catalyst were determined by EDS (energy dispersive X-ray spectroscopy) performed in SEM. Thermal analysis (TG-DTG) was carried out using an STA 1500 Rheometric Scientific (England). The flow rate of air was 120 mL min^−1^ and the ramping rate of the sample was 2 °C min^−1^. VSM measurements were recorded using a SQUID magnetometer at 298 K (Quantum Design MPMS XL). Melting points were taken in open capillaries using BUCHI 510 melting point apparatus and are uncorrected.

### General protocol for the synthesis of the {Fe_3_O_4_@SiO_2_@(CH_2_)_3_5-phenyl-1*H*-tetrazole-SO_3_H/Cl}

Firstly, in a 250 mL flask, 1.0 g of Fe_3_O_4_, 20.0 mL of H_2_O, 3.0 mL of NH_3_, 80.0 mL of EtOH and 3.0 mL of tetraethylorthosilicate (TEOS) were mixed and refluxed to afford silica-coated Fe_3_O_4_ (Fe_3_O_4_@SiO_2_). In the next step, 3.0 g of Fe_3_O_4_@SiO_2_, 10.0 mmol of (3-chloropropyl)trimethoxysilane and 80.0 mL of dry toluene were refluxed under nitrogen for 12 h. The as-prepared Fe_3_O_4_@SiO_2_@(CH_2_)_3_Cl was separated with an external magnet, washed with dry toluene and anhydrous diethyl ether, and dried at 80 °C for 6 h under vacuum. Then 5-phenyl-1*H*-tetrazole (0.73 g, 5.0 mmol) and K_2_CO_3_ (0.69 g, 5.0 mmol) in 50.0 mL of DMF were added to the Fe_3_O_4_@SiO_2_@(CH_2_)_3_Cl and the mixture was refluxed for 24 h. The resulting solid was filtered, washed with ethanol and distilled water and dried at 80 °C for 6 h under vacuum. Finally, chlorosulfonic acid (1.165 g, 10.0 mmol) was added dropwise to the Fe_3_O_4_@SiO_2_@(CH_2_)_3_5-phenyl-1*H*-tetrazole in dry dichloromethane and the mixture was stirred for 3 h. Then with similar steps of filtering, washing and drying, {Fe_3_O_4_@SiO_2_@(CH_2_)_3_5-phenyl-1*H*-tetrazole-SO_3_H/HCl} as a magnetically recoverable catalyst was obtained.

### General protocol for the one-pot synthesis of 1-carbamoyl-1-phenylureas

In a typical procedure, to a mixture of arylcyanamide (1.0 mmol) and sodium cyanate (0.065 g, 1.0 mmol) 0.2 g of the {Fe_3_O_4_@SiO_2_@(CH_2_)_3_5-phenyl-1*H*-tetrazole-SO_3_H/Cl} catalyst in 10.0 mL of H_2_O was added and stirred under reflux conditions for an appropriate amount of time. The reaction progress was monitored using TLC. After completion of the reaction, the catalyst was recovered magnetically and the mixture was cooled for the product to precipitate. All of the products were purified *via* recrystallization from EtOAC–*n*-hexane. The products were obtained in high yields and characterized by FT-IR, ^1^H NMR, ^13^C NMR, CHN and melting points.

#### 1-Carbamoyl-1-(3-bromophenyl)urea (1)

FT-IR (KBr, cm^−1^) 3294, 3249, 3112, 3181, 3077, 1686, 1665, 1592, 1542, 1474, 1418, 1372, 1334, 1311, 1281, 1258, 1225, 1167, 1092, 1067, 996, 964, 889, 873, 746, 680, 657, 608, 535, 435; ^1^H NMR (400 MHz, CDCl_3_) *δ* 7.79 (s, 1H), 7.50 (s, br, 4H), 7.43 (d, *J* = 7.6 Hz, 1H), 7.26 (d, *J* = 8.0 Hz, 1H), 7.19 (t, *J* = 7.8 Hz, 1H); ^13^C NMR (100 MHz, CDCl_3_) *δ* 168.5, 139.1, 130.3, 127.3, 122.8, 122.6, 118.3; anal. calcd for C_8_H_8_BrN_3_O_2_: C, 37.23; H, 3.12; N, 16.28. Found: C, 37.31; H, 3.20; N, 16.36.

#### 1-Carbamoyl-1-(4-chlorophenyl)urea (2)

Mp = 178–180 °C; FT-IR (KBr, cm^−1^) 3304, 3193, 3127, 1665, 1068, 1539, 1490, 1394, 1371, 1291, 1260, 1170, 1090, 1009, 968, 830, 750, 708, 607, 506, 450; ^1^H NMR (400 MHz, CDCl_3_) *δ*_H_ = 7.48 (d, *J* = 8.0 Hz, 2H), 7.39 (s, br, 4H), 7.30 (d, *J* = 8.0 Hz, 2H); ^13^C NMR (100 MHz, CDCl_3_) *δ*_C_ = 168.3, 136.4, 129.3, 129.0, 121.1; anal. calcd for C_8_H_8_N_3_O_2_Cl: C, 44.98; H, 3.77; N, 19.67. Found: C, 44.91; H, 3.69; N, 19.72.

#### 1-Carbamoyl-1-(4-methylphenyl)urea (3)

Mp = 154–156 °C; FT-IR (KBr, cm^−1^) 3291, 3188, 3123, 3067, 1662, 1603, 1551, 1510, 1455, 1402, 1365, 1321, 1264, 1114, 1040, 1013, 820, 753, 619, 605, 510; ^1^H NMR (400 MHz, CDCl_3_) *δ*_H_ = 7.71 (s, br, 4H), 7.40 (d, *J* = 8.2 Hz, 2H), 7.13 (d, *J* = 8.2 Hz, 2H), 2.33 (s, 3H); ^13^C NMR (100 MHz, CDCl_3_) *δ*_C_ = 168.3, 136.4, 129.3, 129.0, 121.1; anal. calcd for C_9_H_11_N_3_O_2_: C, 55.95; H, 5.74; N, 21.75. Found: C, 56.02; H, 5.80; N, 21.82.

#### 1-Carbamoyl-1-(4-methoxylphenyl)urea (4)

Mp = 130–132 °C; FT-IR (KBr, cm^−1^) 3277, 3243, 3191, 3131, 3068, 1647, 1606, 1561, 1512, 1465, 1455, 1440, 1411, 1369, 1320, 1303, 1246, 1175, 1113, 1031, 970, 838, 820, 767, 650, 619, 607, 520, 463; ^1^H NMR (400 MHz, CDCl_3_) *δ*_H_ = 7.80 (s, br, 4H), 7.42 (d, *J* = 8.4 Hz, 2H), 6.85 (d, *J* = 8.4 Hz, 2H), 3.79 (s, 3H); ^13^C NMR (100 MHz, CDCl_3_) *δ*_C_ = 168.3, 136.4, 129.3, 129.0, 121.1; anal. calcd for C_9_H_11_N_3_O_3_: C, 51.67; H, 5.30; N, 20.09. Found: C, 51.76; H, 5.37; N, 20.15; MS: *m*/*z* = 203 (M^+^).

#### 1-Carbamoyl-1-(2,4-dimethylphenyl)urea (5)

Mp = 127–129 °C; FT-IR (KBr, cm^−1^) 3277, 3192, 3010, 1652, 1615, 1598, 1531, 1365, 1299, 1281, 1017, 973, 875, 813, 708, 625, 610, 505, 448; ^1^H NMR (400 MHz, CDCl_3_) *δ*_H_ = 7.52 (d, *J* = 8.8 Hz, 1H), 7.11 (br, 4H), 7.04–7.01 (m, 2H), 2.31 (s, 3H), 2.23 (s, 3H); ^13^C NMR (100 MHz, CDCl_3_) *δ*_C_ = 168.6, 135.3, 132.9, 131.2, 130.2, 127.2, 124.1, 20.9, 17.8; anal. calcd for C_10_H_13_N_3_O_2_: C, 57.96; H, 6.32; N, 20.28. Found: C, 58.08; H, 6.41; N, 20.36.

#### 1-Carbamoyl-1-(2,4-dimethoxylphenyl)urea (6)

Mp = 128–130 °C; FT-IR (KBr, cm^−1^) 3285, 3192, 3131, 2999, 2964, 2839, 1663, 1614, 1539, 1504, 1467, 1437, 1417, 1375, 1282, 1258, 1220, 1184, 1127, 1042, 1024, 968, 916, 839, 801, 675, 607, 511; ^1^H NMR (400 MHz, CDCl_3_) *δ*_H_ = 8.23 (d, *J* = 6.0 Hz, 1H), 7.57 (s, br, 4H), 6.50–6.47 (m, 2H), 3.87 (s, 3H), 3.81 (s, 3H); ^13^C NMR (100 MHz, CDCl_3_) *δ*_C_ = 167.9, 156.3, 149.1, 121.3, 120.8, 103.7, 98.6, 55.7, 55.5; anal. calcd for C_10_H_13_N_3_O_4_: C, 50.21; H, 5.48; N, 17.56. Found: C, 50.28; H, 5.53; N, 17.64.

#### 2-(4-(1-Carbamoylureido)phenyl)malonamide (7)

Mp = 303–306 °C; FT-IR (KBr, cm^−1^) 3301, 3169, 3081, 1662, 1571, 1513, 1453, 1402, 1368, 1318, 1038, 1007, 960, 834, 749, 604, 591, 521; ^1^H NMR (400 MHz, DMSO-d_6_) *δ*_H_ = 9.86 (s, br, 8H), 7.46 (s, 4H); ^13^C NMR (100 MHz, DMSO-d_6_) *δ*_C_ = 168.3, 134.9, 119.8; anal. calcd for C_10_H_12_N_6_O_4_: C, 42.86; H, 4.32; N, 29.99. Found: C, 42.91; H, 4.41; N, 30.08.

## Results and discussion

**Scheme 2:**
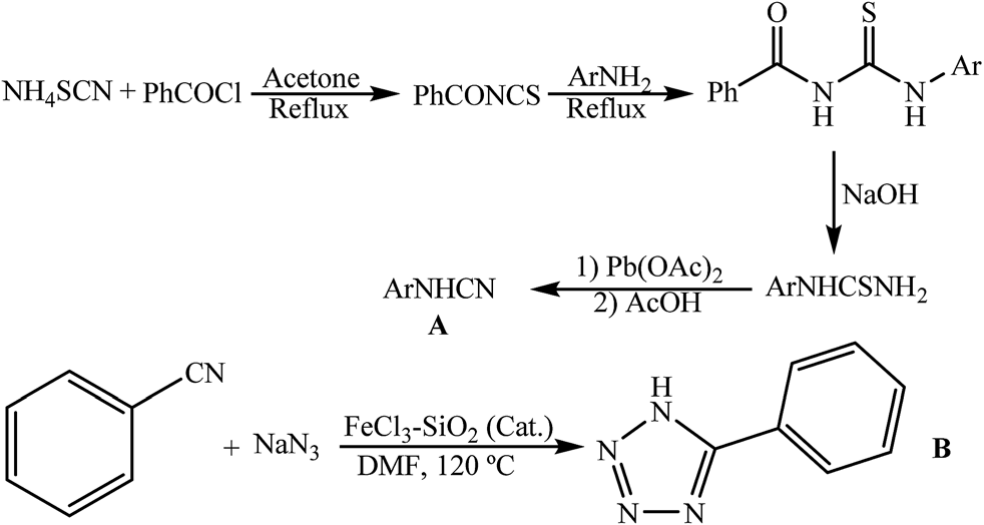
Preparation of arylcyanamides (A) and 5-phenyl-1*H*-tetrazole (B).

Our recent studies have shown that arylcyanamides and tetrazoles could be used as highly active compounds in organic synthesis.^8,15^ However, a lack of convenient methods for the preparation of the arylcyanamides and ionic liquid based tetrazoles strongly restricts their potential application in organic synthesis. In this work, the arylcyanamides and 5-phenyl-1*H*-tetrazole were prepared according to our recent work on aryl amines^15*c*^ and benzonitrile^15*d*^ (Scheme 2).

Due to our interest in green protocols, in the present work, for the first time we have reported the synthesis of 1-carbamoyl-1-phenylureas using [FSTet-SO_3_H]Cl as a magnetic Brønsted acidic ionic liquid catalyst in water as a green solvent.

In this work, by combining the advantages of a Brønsted acidic ionic liquid as a H^+^ source and silica-coated magnetic nanoparticles (Fe_3_O_4_@SiO_2_) with high surface area, excellent thermal stability, low toxicity, and easy synthesis and separation from the reaction mixture by an external magnet, a [FSTet-SO_3_H]Cl nanocomposite has been produced using a simple method. To the best of our knowledge, this is the first report wherein an ionic liquid based tetrazole has been immobilized on the Fe_3_O_4_@SiO_2_ surface as a powerful catalytic support.

### Characterization of the [FSTet-SO_3_H]Cl

**Scheme 3:**
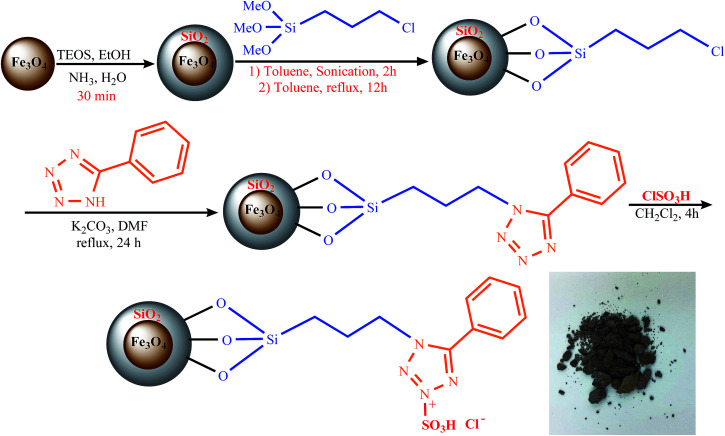
Synthesis of {Fe_3_O_4_@SiO_2_@(CH_2_)_3_5-phenyl-1*H*-tetrazole-SO_3_H/Cl}.

As shown in Scheme 3, the [FSTet-SO_3_H]Cl was prepared in four steps: (1) coating the Fe_3_O_4_ with the silica (Fe_3_O_4_@SiO_2_), (2) linking the Fe_3_O_4_@SiO_2_ with the 3-chloropropyltriethoxysilane, (3) grafting the Fe_3_O_4_@SiO_2_@(CH_2_)_3_Cl with 5-phenyl-1*H*-tetrazole and (4) converting the Fe_3_O_4_@SiO_2_@(CH_2_)_3_5-phenyl-1*H*-tetrazole to an ionic liquid by chlorosulfonic acid. The synthesized catalyst was characterized using FT-IR, XRD, TEM, FE-SEM, EDS, VSM, TGA and DTG.

**Fig. 1:**
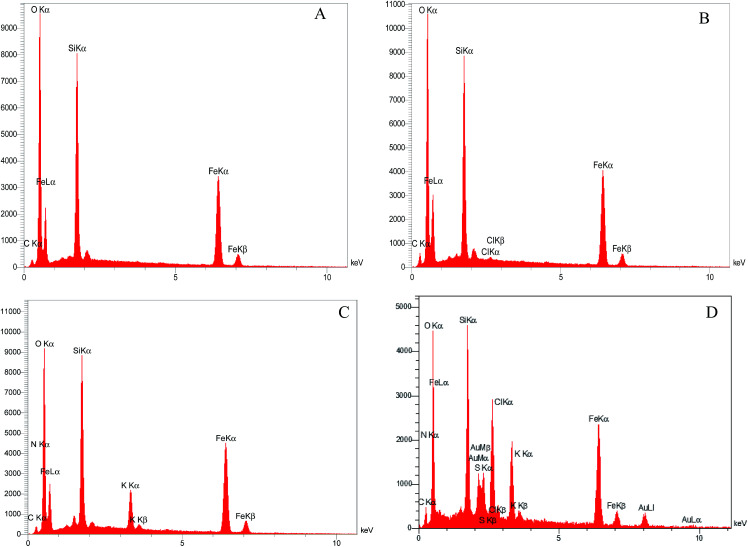
The EDS spectra of Fe_3_O_4_@SiO_2_ (A), Fe_3_O_4_@SiO_2_@(CH_2_)_3_-Cl (B), Fe_3_O_4_@SiO_2_@(CH_2_)_3_5-phenyl-1*H*-tetrazole (C) and [FSTet-SO_3_H]Cl (D).

The chemical composition of Fe_3_O_4_@SiO_2_, Fe_3_O_4_@SiO_2_@(CH_2_)_3_Cl, Fe_3_O_4_@SiO_2_@(CH_2_)_3_5-phenyl-1*H*-tetrazole, and [FSTet-SO_3_H]Cl was analyzed by Energy Dispersive X-ray Spectroscopy (EDS). The EDS spectra of these materials indicate the presence of the corresponding elements in their structure. The EDS spectrum of [FSTet-SO_3_H]Cl confirmed that it was composed of Fe, Si, N, C, O and S (Fig. 1). Fig. 1 confirmed that Fe, Si, N, C, and O were the main components present in both Fe_3_O_4_@SiO_2_@(CH_2_)_3_5-phenyl-1*H*-tetrazole and [FSTet-SO_3_H]Cl along with the S element which is present only in [FSTet-SO_3_H]Cl further demonstrating the formation of an ionic liquid in the case of [FSTet-SO_3_H]Cl.

**Fig. 2:**
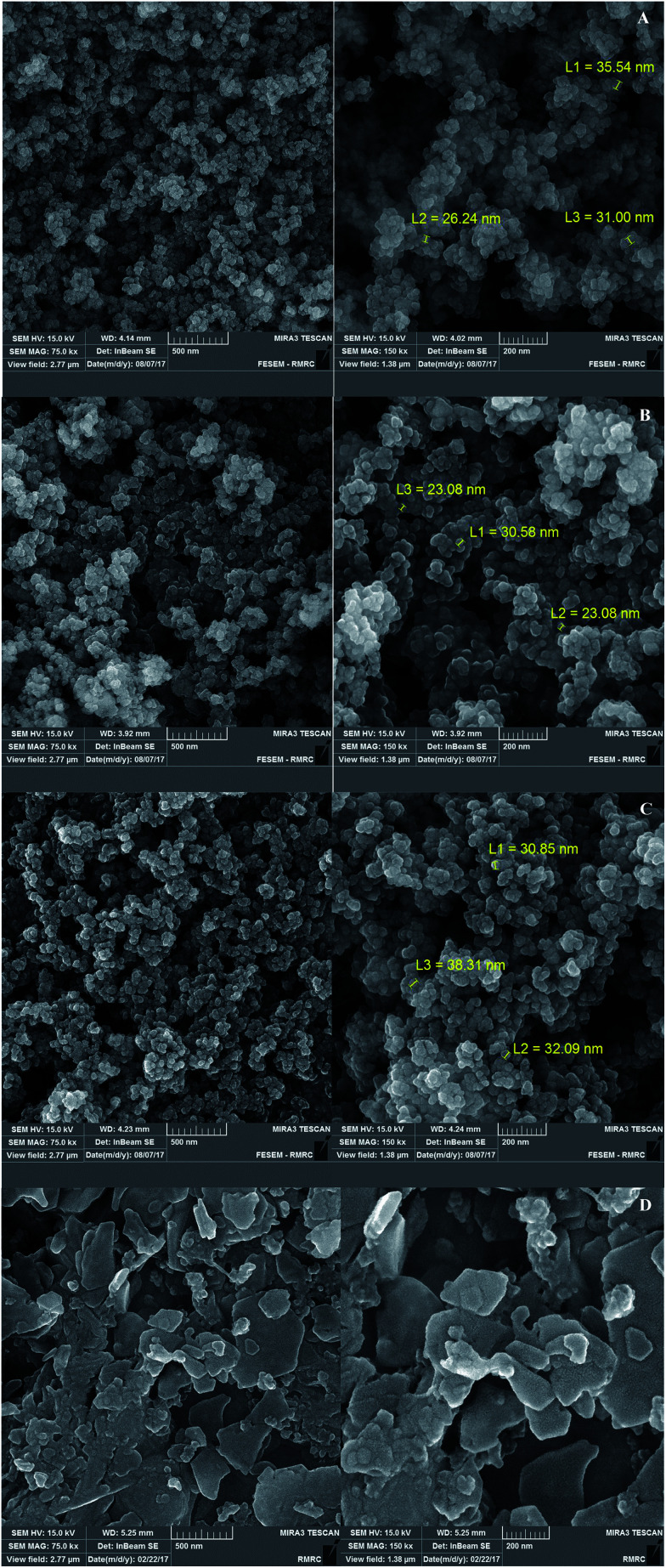
FESEM images of Fe_3_O_4_@SiO_2_ (A), Fe_3_O_4_@SiO_2_@(CH_2_)_3_Cl (B), Fe_3_O_4_@SiO_2_@(CH_2_)_3_5-phenyl-1*H*-tetrazole (C) and [FSTet-SO_3_H]Cl (D).

**Fig. 3:**
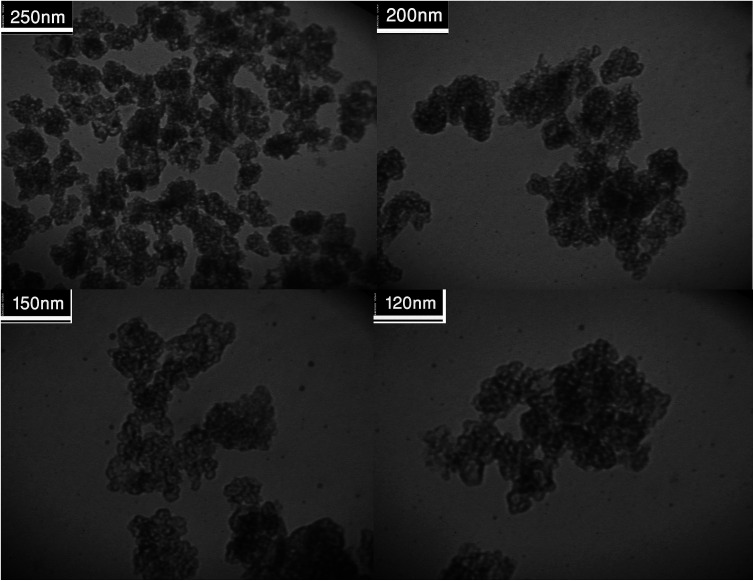
TEM images of [FSTet-SO_3_H]Cl.

Fig. 2 shows the FESEM images of Fe_3_O_4_@SiO_2_, Fe_3_O_4_@SiO_2_@(CH_2_)_3_Cl, Fe_3_O_4_@SiO_2_@(CH_2_)_3_5-phenyl-1*H*-tetrazole and [FSTet-SO_3_H]Cl. These materials show a spherical morphology with an average particle size in the range 23–39 nm. The morphology of [FSTet-SO_3_H]Cl was also studied from TEM images at different magnifications (Fig. 3). The morphology calculated from the FESEM images was in agreement with TEM images in the case of [FSTet-SO_3_H]Cl. However, the average particle size was found to be smaller in the TEM images.

**Fig. 4:**
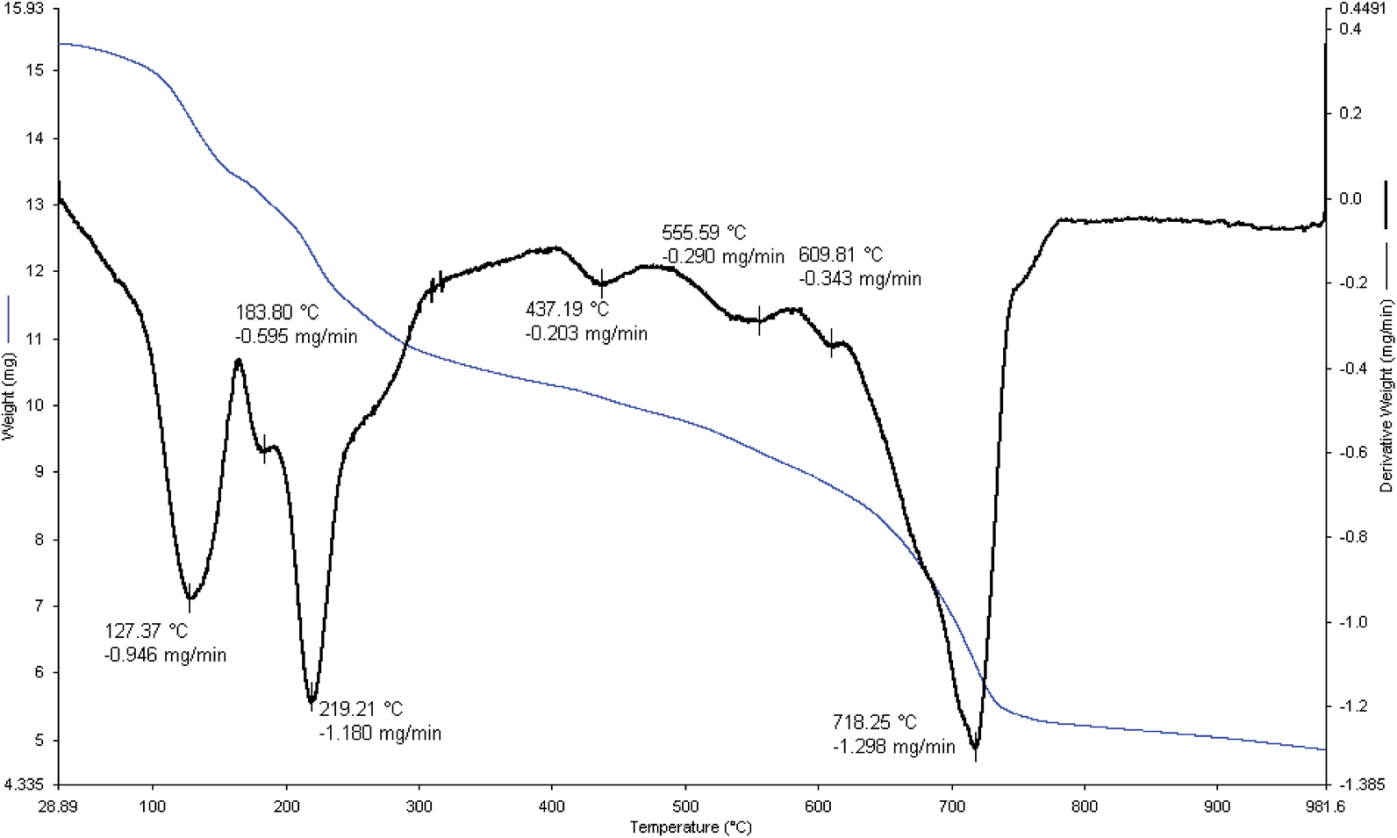
TG-DTA analysis of [FSTet-SO_3_H]Cl.

The thermal stability of [FSTet-SO_3_H]Cl was also analyzed by TGA (thermogravimetric analysis) and DTA (differential thermal analysis) experiments in air flow (120 mL min^−1^) at a heating rate of 2 °C min^−1^ on an autonomic TG-DTA. As shown in Fig. 4, three weight loss stages were observed in air flow for [FSTet-SO_3_H]Cl. The first weight-loss step mainly happened at 120–190 °C and is associated with desorbed water or other organic solvents which were employed during the preparation steps of the catalyst. In the second stage at 218–300 °C, a weight loss is observed which can be attributed to the thermal decomposition of 5-phenyl-1*H*-tetrazole functionalized with chlorosulfonic acid on the surface of the silica coating. Finally, the weight loss observed at 650–750 °C is due to the decomposition of the catalyst.

**Fig. 5:**
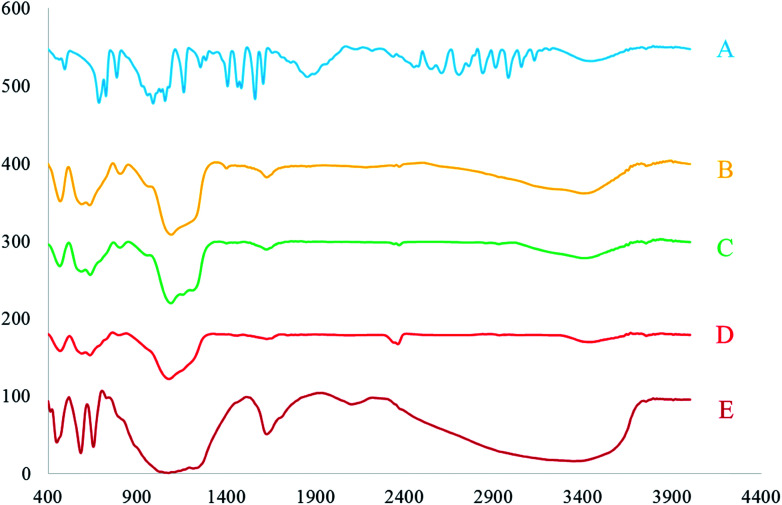
The FT-IR spectra of 5-phenyl-1*H*-tetrazole (A), Fe_3_O_4_@SiO_2_ (B), Fe_3_O_4_@SiO_2_@(CH_2_)_3_Cl (C), Fe_3_O_4_@SiO_2_@(CH_2_)_3_5-phenyl-1*H*-tetrazole (D) and [FSTet-SO_3_H]Cl (E).

FT-IR analysis was carried out to confirm the structure and the formation of [FSTet-SO_3_H]Cl. Fig. 5 shows the FT-IR spectra of 5-phenyl-1*H*-tetrazole (A), Fe_3_O_4_@SiO_2_ (B), Fe_3_O_4_@SiO_2_@(CH_2_)_3_Cl (C), Fe_3_O_4_@SiO_2_@(CH_2_)_3_5-phenyl-1*H*-tetrazole (D) and [FSTet-SO_3_H]Cl (E). The structure of 5-phenyl-1*H*-tetrazole was in agreement with the FT-IR spectra data. The disappearance of one strong and sharp absorption band (CN stretching band) in benzonitrile, and the appearance of an NH stretching band in the FT-IR spectroscopy, was evidence for the formation of 5-phenyl-1*H*-tetrazole (Fig. 5A). In Fig. 5B–E, the peak at 3600–3100 cm^−1^ is due to the O–H stretching mode. The appearance of two peaks at 570 cm^−1^ and 617 cm^−1^ (Fig. 5B) supports the formation of Fe_3_O_4_. The absorption peak at 570 cm^−1^ is attributed to Fe–O vibration of the Fe_3_O_4_ MNPs. The formation of Fe_3_O_4_@SiO_2_ was confirmed by the appearance of peaks at 450, 801, 955 and 1097 cm^−1^ which are assigned to the Si–O–Si bending, Si–O bending, Si–OH stretching and Si–O–Si stretching, respectively (Fig. 5B). The peak at 1619 cm^−1^ may be attributed to C=C and C=N vibrations of the tetrazolium moiety of the ionic liquid (Fig. 5E).

**Fig. 6:**
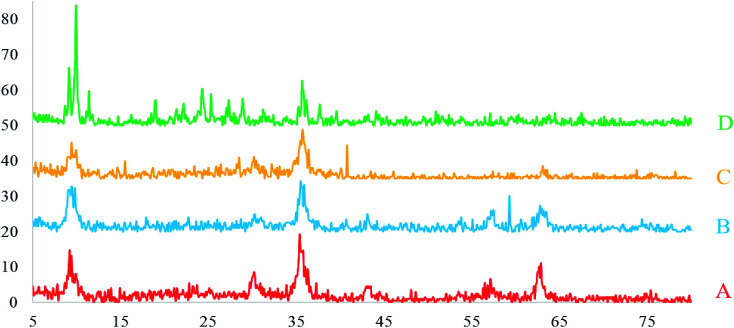
The XRD patterns of Fe_3_O_4_@SiO_2_ (A), Fe_3_O_4_@SiO_2_@(CH_2_)_3_Cl (B), Fe_3_O_4_@SiO_2_@(CH_2_)_3_5-phenyl-1*H*-tetrazole (C) and [FSTet-SO_3_H]Cl (D).

To determine the crystallographic structure of Fe_3_O_4_@SiO_2_, Fe_3_O_4_@SiO_2_@(CH_2_)_3_Cl, Fe_3_O_4_@SiO_2_@(CH_2_)_3_5-phenyl-1*H*-tetrazole and [FSTet-SO_3_H]Cl, XRD analysis was carried out (Fig. 6). As shown in Fig. 6, peaks of 2*θ* at 30.00°, 35.30°, 42.80°, 53.00°, 56.80° and 62.50° can be indexed as (*hkl*) to the (220), (311), (400), (422), (511) and (440) planes of cubic iron oxide.

**Fig. 7:**
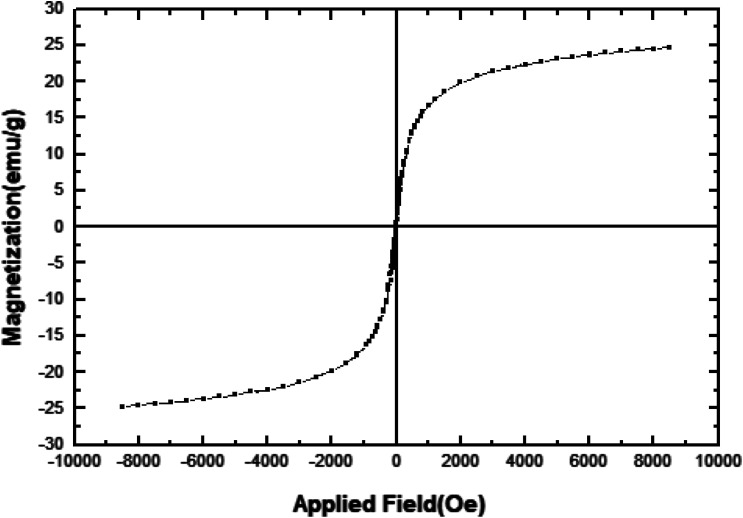
Magnetization curve for [FSTet-SO_3_H]Cl.

In order to study the magnetic properties of [FSTet-SO_3_H]Cl, a vibrating sample magnetometer (VSM) was used to characterize the as-prepared catalyst with a magnetometer at 298 K and with field sweeping from −10 000 to +10 000 Oe. Fig. 7 shows a typical room temperature magnetization curve of [FSTet-SO_3_H]Cl. As demonstrated in Fig. 7, the magnetization curves of the as-prepared ionic liquid display no hysteresis loop which demonstrates its superparamagnetic characteristics. Therefore, at the end of the reaction, [FSTet-SO_3_H]Cl could simply be collected from the reaction mixture using an external magnet.

**Scheme 4:**
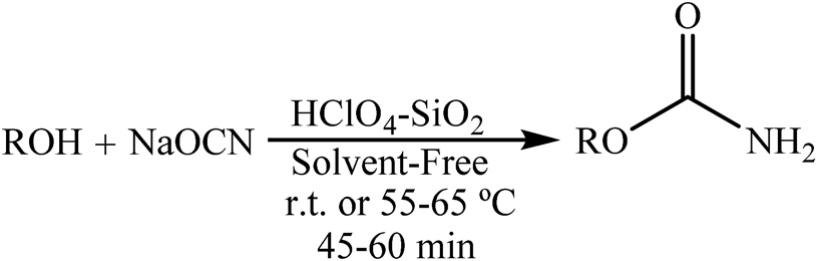
Preparation of primary carbamates.

Following the synthesis of 1-arylureas^8^ and in a further study on the preparation of nitrogen-containing compounds, we focussed on the synthesis of 1-carbamoyl-1-phenylureas as nitrogen-rich compounds from arylcyanamides and sodium cyanate (NaOCN) as starting materials. In 2008, our research group reported the synthesis of primary carbamates from the reaction between phenol or alcohols with NaOCN in the presence of HClO_4_–SiO_2_ at room temperature or 55–65 °C for an appropriate time in high yields and under solvent-free conditions (Scheme 4).^16^

Herein, in the course of our research on the synthesis of the nitrogen-containing compounds,^15^ we now wish to report the preparation of novel 1-carbamoyl-1-phenylureas by the reaction of NaOCN with arylcyanamides in the presence of a [FSTet-SO_3_H]Cl catalyst in water under reflux conditions. Apparently, to the best of our knowledge, so far no methodology has been reported where NaOCN is used as an effective salt in the synthesis of the 1-carbamoyl-1-phenylureas.

We applied our catalyst to the synthesis of 1-carbamoyl-1-phenylureas under reflux conditions and 3-bromophenylcyanamide was chosen as a model substrate. Various reaction conditions including the amount of the [FSTet-SO_3_H]Cl catalyst and temperature were varied to examine the influence on the compositions in the reaction mixture (Table 1). We observed that 1-(3-bromophenyl)-1-carbamoylurea (1) was not produced without using the [FSTet-SO_3_H]Cl catalyst (Table 1, entry 1). However, with the presence of a 3-bromophenylcyanamide, NaOCN, [FSTet-SO_3_H]Cl catalyst and H_2_O mixture at room temperature, product (1) was obtained in 23% yield. We found that the yield and reaction rate were improved significantly by increasing the reaction temperature. In general, the best result was obtained with 0.2 g of the [FSTet-SO_3_H]Cl catalyst under reflux conditions (Table 1, entry 4), whereas no significant improvements in the reaction yield and time were observed by further increasing the amount of the [FSTet-SO_3_H]Cl catalyst from 0.1 g to 0.3 g (Table 1, entry 5).

**Table 1:** Synthesis of the 1-(3-bromophenyl)-1-carbamoylurea (1): reaction condition optimization^a^

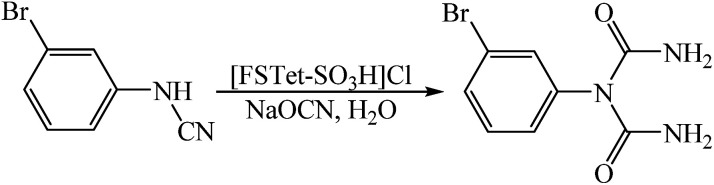				
Entry	Cat. (g)	Temperature (°C)	Time (h)	Yield^b^ (%)
1	0.0	Reflux	5	0.0
2	0.05	Reflux	10	43
3	0.1	Reflux	7	70
4	0.2	Reflux	5	90
5	0.3	Reflux	5	90
6	0.2	R.T.	10	23

a The reactions were performed with 1.0 mmol of 3-bromophenylcyanamide and 1.0 mmol NaOCN in H_2_O (10 mL).

b Yield refers to the pure isolated product.

With the optimized conditions in hand, we next tested the substrate scope of this transformation. Excellent yields could be achieved regardless of the substituents associated with the arylcyanamides (Table 2). Different substituents such as Br, Cl, OMe and Me groups were compatible, and achieved a yield up to 90% (1–7). Both electron-withdrawing and electron-donating groups in the cyanamides were compatible. As shown in Table 2, the arylcyanamides with electron-donating groups were completed under reflux conditions after 3 h and the corresponding products were obtained in shorter reaction times because of their greater ability to attack the NaOCN. However, the species bearing electron withdrawing groups required higher reaction times. As shown in Table 2, 1,4-phenylenecyanamide interestingly afforded the double-addition product (7), due to presence of two CN groups. The products were characterized by IR, ^1^H NMR and ^13^C NMR spectroscopy, elemental analysis and melting point determination.

**Table 2:** Substrate scope^a^

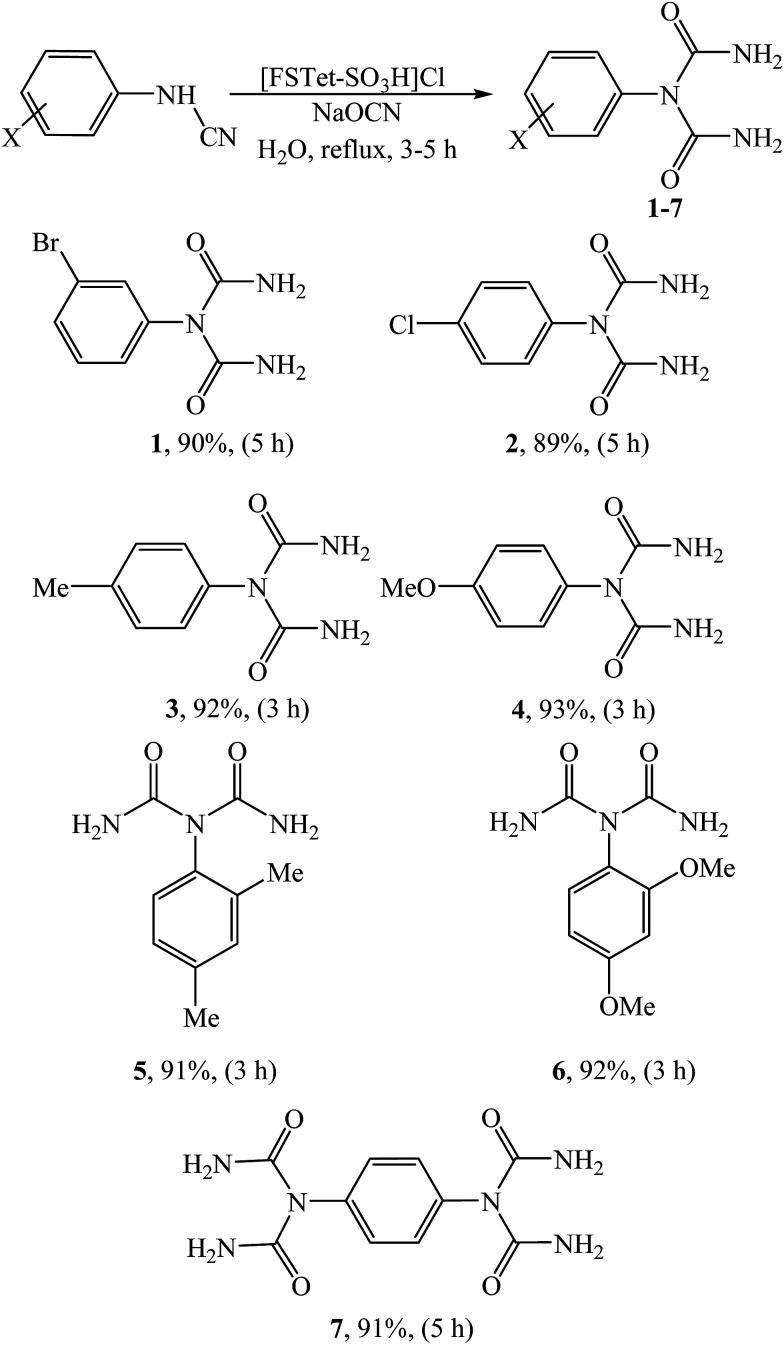

a The reactions were performed with 1.0 mmol arylcyanamide, 1.0 mmol NaOCN and 0.2 g [FSTet-SO_3_H]Cl catalyst in H_2_O (10 mL) under reflux conditions. Yield refers to the pure isolated product.

**Fig. 8:**
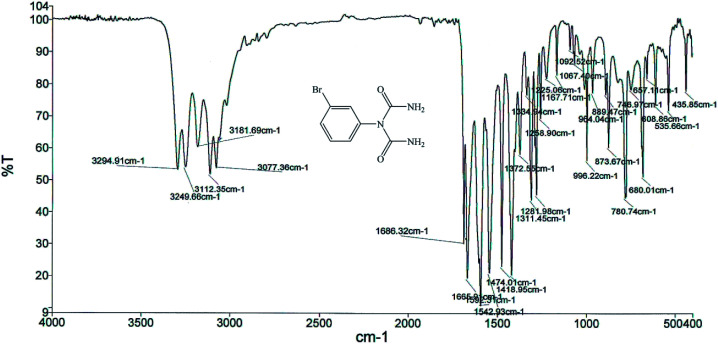
FT-IR spectrum of 1-carbamoyl-1-(3-bromophenyl)urea (1).

**Fig. 9:**
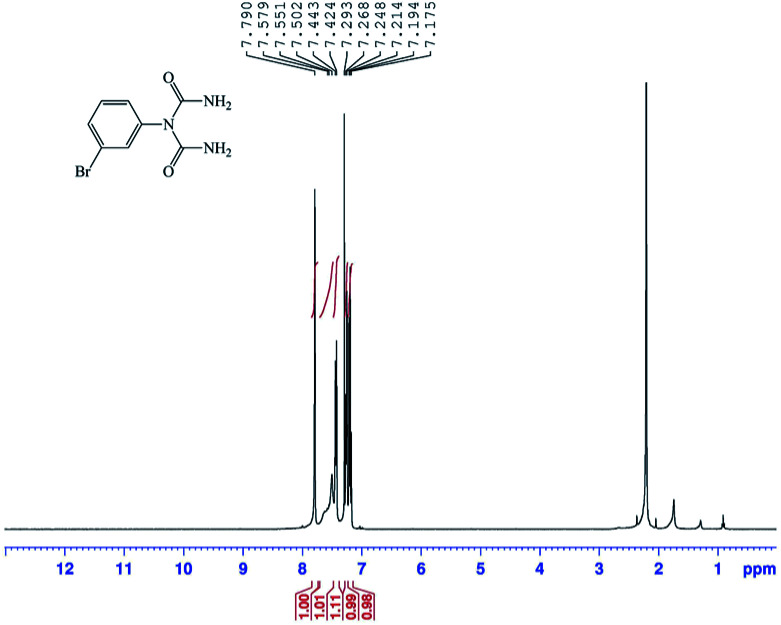
Expanded ^1^H NMR spectrum (400 MHz, CDCl_3_) of 1-carbamoyl-1-(3-bromophenyl)urea (1).

**Fig. 10:**
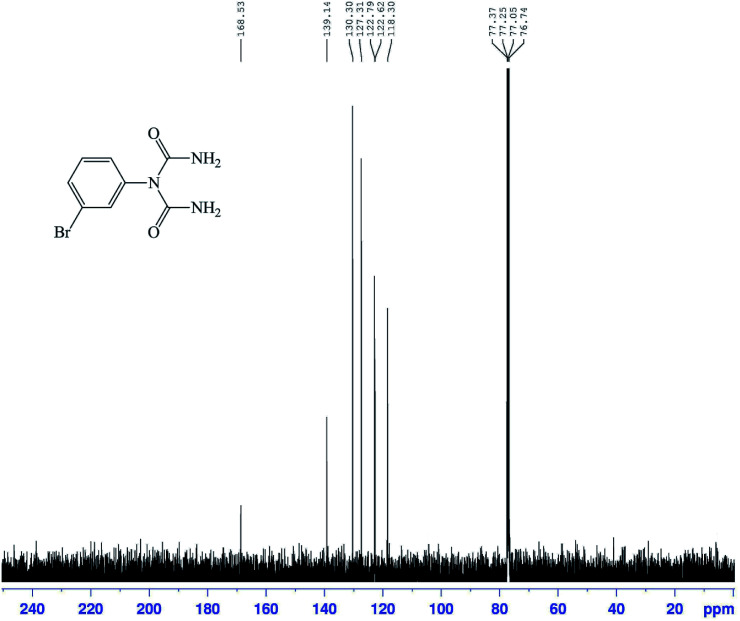
^13^C NMR spectrum (100 MHz, CDCl_3_) of 1-carbamoyl-1-(3-bromophenyl)urea (1).

The structures of the 1-carbamoyl-1-phenylureas were in agreement with their FT-IR and NMR spectra. In the FT-IR spectra of the 1-carbamoyl-1-phenylureas, three new peaks appeared corresponding to C=O and NH_2_ groups absorption vibrations and the CN peak had disappeared (Fig. 8). The ^1^H NMR spectra showed one characteristic peak belonging to the NH_2_ group (Fig. 9). The appearance of a carbon signal corresponding to a carbonyl group in the ^13^C NMR spectra is evidence of the formation of 1-carbamoyl-1-phenylureas (Fig. 10).

**Scheme 5:**
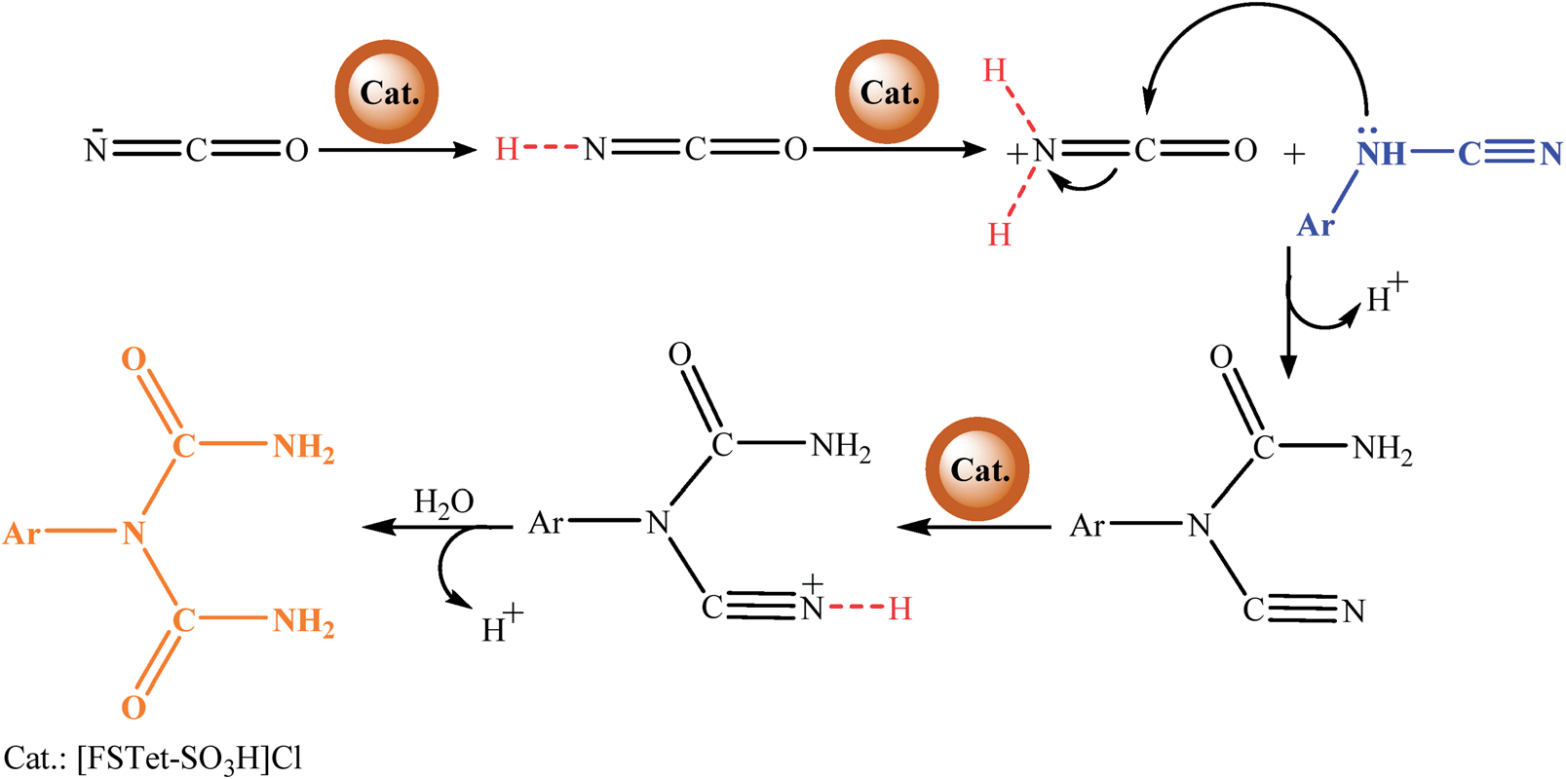
Possible mechanism for the synthesis of 1-carbamoyl-1-phenylureas.

The possible mechanism for the synthesis of 1-carbamoyl-1-phenylureas under reflux conditions is shown in Scheme 5. The [FSTet-SO_3_H]Cl catalyst as a Brønsted acidic ionic liquid has an important role in the synthesis of 1-carbamoyl-1-phenylureas. [FSTet-SO_3_H]Cl is supposed to convert the ^−^N=C=O to a ^+^H_2_N=C=O electrophile and enhance its reactivity with cyanamide as a nucleophile. In the first step, the presence of the [FSTet-SO_3_H]Cl catalyst leads to the protonation of sodium cyanate. Next, arylcyanamides attack the protonated cyanate through a pair of nitrogen electrons. Eventually, due to the presence of the [FSTet-SO_3_H]Cl catalyst and water in the reaction medium, 1-aryl-1-cyanourea was hydrolyzed and the final product was produced.

**Fig. 11:**
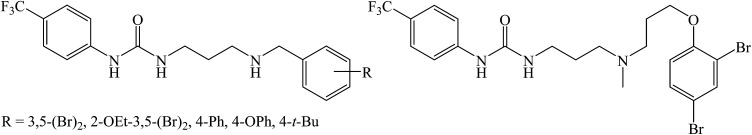
Chemical structures of many bioactive arylureas.

They are important structural motifs for many arylurea analogs as broad-spectrum antibacterial agents (Fig. 11).^17^ Nevertheless, there are no reports on the synthesis of 1-carbamoyl-1-phenylureas and investigations of their antibacterial properties. In the present study, for the first time, we report the antibacterial properties of 2-(4-(1-carbamoylureido)phenyl)malonamide (7) as a model substrate.

*Escherichia coli* is a common inhabitant of the intestinal tract of humans and warm-blooded animals. Most strains of *E. coli* are harmless and are a part of the normal intestinal microflora. These strains serve a useful function in the body by suppressing the growth of harmful bacteria and by synthesizing appreciable amounts of vitamins. However, several pathogenic *E. coli* strains have emerged which cause disease in humans. Pathogenic *E. coli* can be divided into intestinal pathogens causing diarrhoea, and extra intestinal *E. coli* causing a variety of infections in both humans and animals.^18^ Through this report we investigated the antibacterial activity of synthesized 2-(4-(1-carbamoylureido)phenyl)malonamide (7) against pathogenic *E. coli* as following.

**Fig. 12:**
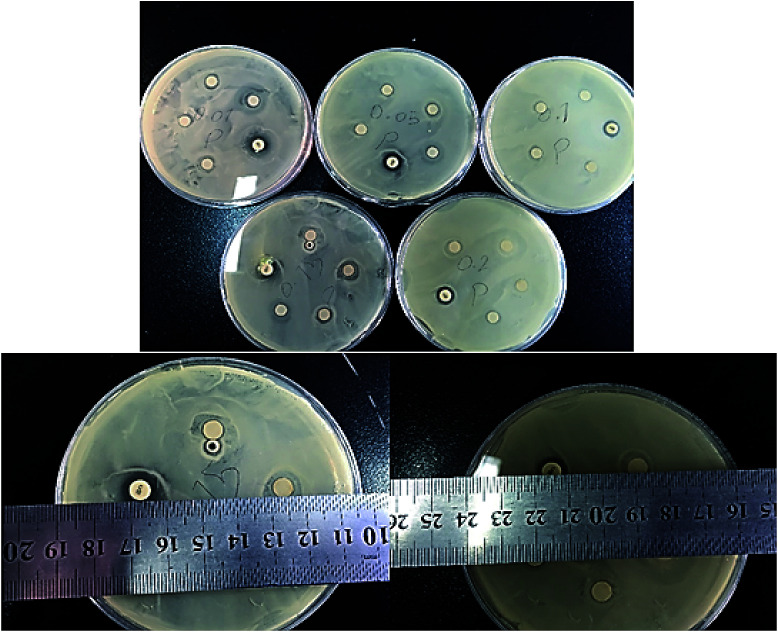
Antibacterial activity of 2-(4-(1-carbamoylureido)phenyl)malonamide (7) against *E. coli*.

The antibacterial activity of the sample was studied against *Escherichia coli* bacteria by disk diffusion method using a Muller Hinton agar culture. The concentrations used for investigations of the sample were 1% (10 mg mL^−1^), 5% (50 mg mL^−1^), 10% (100 mg mL^−1^), 15% (150 mg mL^−1^) and 20% (200 mg mL^−1^) respectively. The results were compared with chloramphenicol as a positive control. For reporting the results of the antibiogram test the minimum protection zone per millimeter (mm) was reported for each test. Furthermore, the frequency of the test was in triplicate for each concentration of the sample. According to Table 3 and Fig. 12, the sample demonstrated no antibacterial activity against *E. coli* in concentrations lower than 15% but showed an antibacterial activity with good potential for concentrations equal and greater than 15%.

**Table 3:** The antibacterial activity of 2-(4-(1-carbamoylureido)phenyl)malonamide (7) on *E. coli*^a^

Compound name	20 mg mL^−1^	15% mg mL^−1^	10% mg mL^−1^	5% mg mL^−1^	1% mg mL^−1^
2-(4-(1-Carbamoylureido)phenyl)malonamide	*t*_1_: 11 mm	*t*_1_: 9 mm	*t*_1_: no result	*t*_1_: no result	*t*_1_: no result
	*t*_2_: 9 mm	*t*_2_: 11 mm	*t*_2_: no result	*t*_2_: no result	*t*_2_: no result
	*t*_3_: 9 mm	*t*_3_: 14 mm	*t*_3_: no result	*t*_3_: no result	*t*_3_: no result
	C^+^: 12 mm	C^+^: 12 mm	C^+^: 12 mm	C^+^: 12 mm	C^+^: 12 mm

a C^+^: chloramphenicol disc diameter (positive control), *t*: test frequency.

As shown from Fig. 12, for both concentrations of 15% and 20% the sample showed a suitable antibacterial activity but in concentrations lower than 15% no antibacterial results were detected. Therefore, the study confirmed that the concentration of the compound is an important factor concerning its biological activity against *E. coli*, and in concentrations greater than 15% the sample demonstrated a good antibacterial activity against the mentioned bacteria compared to the positive control.

**Fig. 13:**
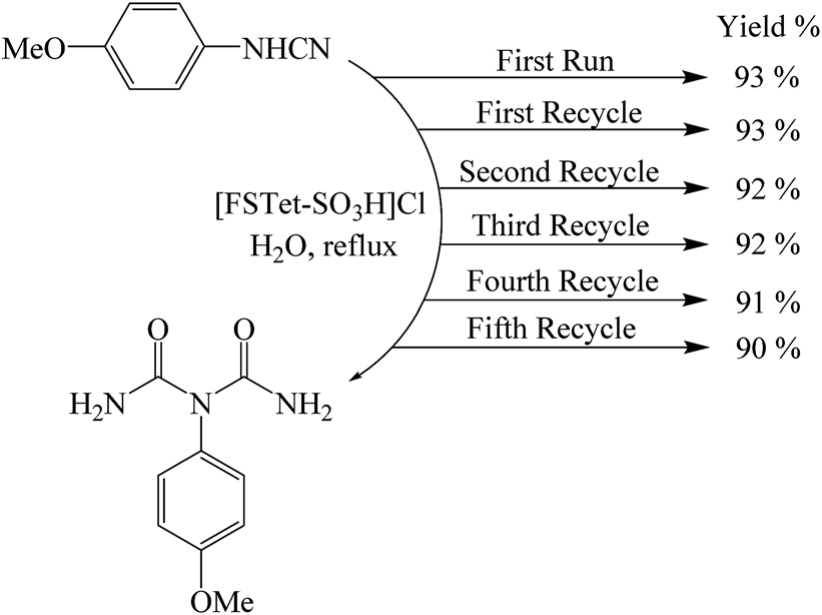
Reusability of [FSTet-SO_3_H]Cl for the synthesis of 1-carbamoyl-1-(4-methoxylphenyl)urea.

**Fig. 14:**
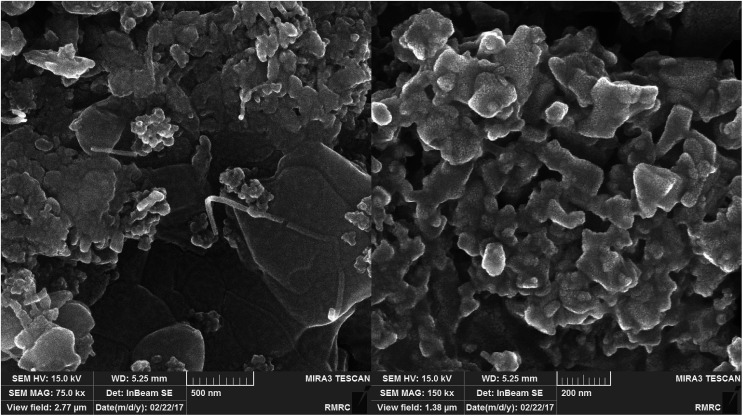
FESEM images of recovered [FSTet-SO_3_H]Cl.

**Fig. 15:**
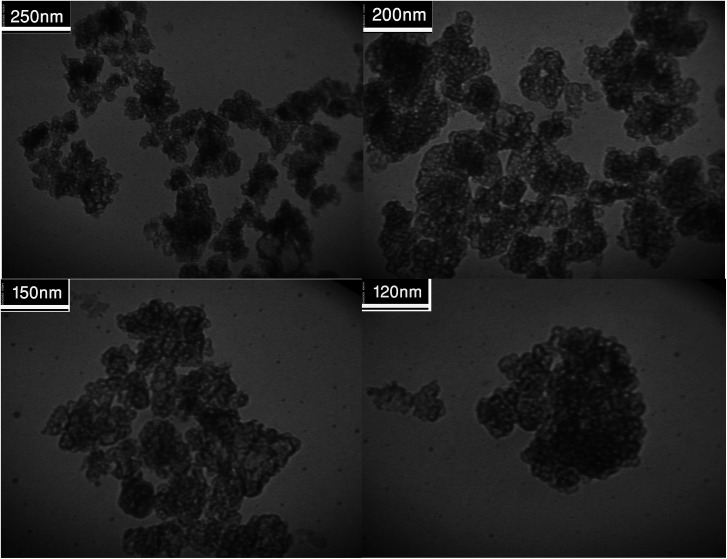
TEM images of recovered [FSTet-SO_3_H]Cl.

One of the most important points in the area of nanocatalysis is the stability and recyclability of heterogeneous catalysts. In order to show the effectiveness of [FSTet-SO_3_H]Cl, catalyst recycling experiments were carried out using 1-(4-methoxyphenyl)urea as the model substrate under optimized conditions. After each cycle, [FSTet-SO_3_H]Cl was separated with an external magnet, washed with ethanol, dried and then reused at least five times without significant loss of catalytic activity (Fig. 13). Easy separation and reusability of the catalysts is one of the most important benefits. As shown in FE-SEM and TEM images of the recycled catalyst (Fig. 14 and 15), no obvious change in the morphology of [FSTet-SO_3_H]Cl was observed.

## Conclusion

In conclusion, we have developed a novel and highly efficient protocol for the preparation of a heterogeneous and recyclable magnetic Brønsted acidic ionic liquid catalyst using 5-phenyl-1*H*-tetrazole. To the best of our knowledge, this is the first report on the synthesis of a tetrazole-based ionic liquid stabilized on the surface of silica-coated magnetic nanoparticles using 3-chloropropyltriethoxysilane as a linker. In this work, we have developed a new strategy for the synthesis of 1-carbamoyl-1-phenylureas from arylcyanamides *via* a one-pot procedure in aqueous media under reflux conditions. A wide range of substituted arylcyanamides as substrates were employed and afforded the desired products in excellent yields. The high yield of products, the efficiency, generality, short reaction time, clean reaction profile, the use of water as a green solvent, the use of a relatively inexpensive catalyst, simplicity and easy work-up procedure, recyclability and reusability of the catalyst, and the straightforward isolation of the products are the advantages of this protocol. The wide substrate scope, green reaction conditions, high yield of products and recovery and recyclability of the catalyst offer the potential for scale-up in pharmaceutical applications. Further studies and the development of other methodologies for the arylcyanamides’ and 1-carbamoyl-1-phenylureas’ reactivities are in progress.

## Conflicts of interest

There are no conflicts to declare.

## Supplementary Material

## References

[cit1] Marsh K. N., Boxall J. A., Lichtenthaler R. (2004). Fluid Phase Equilib..

[cit2] Bentivoglio G., Röder T., Fasching M., Buchberger M., Schottenberger H., Sixta H. (2006). Lenzinger Ber..

[cit3] Welton T. (1999). Chem. Rev..

[cit4] Jonathan G. H., Heather D. W., Richard P. S., Ann E. V., Robin D. R. (1998). Chem. Commun..

[cit5] Pham T. P. T., Cho C. W., Yun Y. S. (2010). Water Res..

[cit6] Yan Q., Wang Y., Zhang H., Xu K., Wei X., Xu P., Zhou Y. (2017). Talanta.

[cit7] Jiang Y., Guo C., Xia H., Mahmood I., Liu C., Liu H. (2009). J. Mol. Catal. B: Enzym..

[cit8] Issaabadi Z., Nasrollahzadeh M., Sajadi S. M. (2017). J. Colloid Interface Sci..

[cit9] Gondi V. B., Gravel M., Rawal V. H. (2005). Org. Lett..

[cit10] Cole A. C., Jensen J. L., Ntai I., Tran K. L. T., Weaver K. J., Forbes D. C., Davis J. H. (2002). J. Am. Chem. Soc..

[cit11] Fraga-Dubreuil J., Bourahla K., Rahmouni M., Bazureau J. P., Hamelin J. (2002). Catal. Commun..

[cit12] Jung S. H., Kohn H. (1985). J. Am. Chem. Soc..

[cit13] Schade D., Topker-Lehmann K., Kotthaus J., Clement B. (2008). J. Org. Chem..

[cit14] Tao G.-H., Guo Y., Joo Y.-H., Twamley B., Shreeve J. M. (2008). J. Mater. Chem..

[cit15] Sajjadi M., Nasrollahzadeh M., Sajadi S. M. (2017). J. Colloid Interface Sci..

[cit16] Modarresi-Alam A. R., Khamooshi F., Nasrollahzadeh M., Amirazizi H. A. (2008). Tetrahedron.

[cit17] Seth P. P., Ranken R., Robinson D. E., Osgood S. A., Risen L. M., Rodgers E. L., Migawa M. T., Jefferson E. A., Swayze E. E. (2004). Bioorg. Med. Chem. Lett..

[cit18] Ahmed N., Dobrindt U., Hacker J., Hasnain S. E. (2008). Nat. Rev. Microbiol..

